# Chromium (VI)‐induced ALDH1A1/EGF axis promotes lung cancer progression

**DOI:** 10.1002/ctm2.1136

**Published:** 2022-12-11

**Authors:** Xin Ge, Mengdie Li, Guo‐Xin Song, Zhixiang Zhang, Jianxing Yin, Zehe Ge, Zhumei Shi, Ling‐Zhi Liu, Bing‐Hua Jiang, Xu Qian, Hua Shen

**Affiliations:** ^1^ Department of Nutrition and Food Hygiene Center for Global Health School of Public Health Nanjing Medical University Nanjing Jiangsu China; ^2^ Jiangsu Key Lab of Cancer Biomarkers, Prevention and Treatment Jiangsu Collaborative Innovation Center for Cancer Personalized Medicine Nanjing Medical University Nanjing Jiangsu China; ^3^ The Key Laboratory of Modern Toxicology of Ministry of Education Nanjing Medical University Nanjing Jiangsu China; ^4^ Department of Pathology The First Affiliated Hospital of Nanjing Medical University Nanjing China; ^5^ Department of Neurosurgery The First Affiliated Hospital of Nanjing Medical University Nanjing Jiangsu China; ^6^ Department of Pathology Anatomy and Cell Biology Department of Medical Oncology Thomas Jefferson University Philadelphia Pennsylvania USA; ^7^ The Academy of Medical Science Zhengzhou University Zhengzhou 450000 China; ^8^ Department of Oncology The First Affiliated Hospital of Nanjing Medical University Nanjing Jiangsu China; ^9^ Department of Oncology Sir Run Run Hospital Nanjing Medical University Nanjing Jiangsu China

**Keywords:** ALDH1A1, cancer stem cell, hexavalent chromium, lung squamous carcinoma

## Abstract

Cr(VI) is broadly applied in industry. Cr(VI) exposure places a big burden on public health, thereby increasing the risk of lung squamous cell carcinoma (LUSC). The mechanisms underlying Cr(VI)‐induced LUSC remain largely elusive. Here, we report that the cancer stem cell (CSC)/tumour‐initiating cell (TIC)‐like subgroup within Cr(VI)‐transformed bronchial epithelial cells (CrT) promotes lung cancer tumourigenesis. Mechanistically, Cr(VI) exposure specifically increases the expression levels of aldehyde dehydrogenase 1A1 (ALDH1A1), a CSC marker, through KLF4‐mediated transcription. ALDH1A1 maintains self‐renewal of CrT/TICs and facilitates the expression and secretion of EGF from CrT/TICs, which subsequently promotes the activation of EGFR signalling in differentiated cancer cells and tumour growth of LUSC. In addition, the ALDH1A1 inhibitor A37 and gemcitabine synergistically suppress LUSC progression. Importantly, high ALDH1A1 expression levels are positively correlated with advanced clinical stages and predict poor survival in LUSC patients. These findings elucidate how ALDH1A1 modulates EGF secretion from TICs to facilitate LUSC tumourigenesis, highlighting new therapeutic strategies for malignant lung cancers.

## Graphical abstract

- CrT/TICs maintained by ALDH1A1 drive lung cancer tumourigenesis within Cr (VI)‐induced cellular malignant transformation.
- Cr(VI)‐exposure specifically induces ALDH1A1 expression via KLF4‐mediated transcription.
- KLF4/ALDH1A1 regulatory axis promotes differentiated cancer cells and tumour growth of LUSC by facilitating EGF expression and secretion from CrT/TICs.
- A37, the ALDH1A1 inhibitor, sensitises LUSC to gemcitabine treatment.

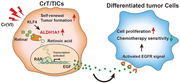

## 1 INTRODUCTION

Hexavalent chromium [Cr(VI)] is listed as a human carcinogen by the International Agency for Research on Cancer. Due to the extensive use in industry, air pollution and occupational exposure to Cr(VI) are placing an increasing burden on public health.^1^ After inhalation, Cr(VI) particles are deposited at the bronchial epithelium, where they remain for years, promoting tumourigenesis of lung squamous cell carcinoma (LUSC).^2^, ^3^ The bronchial epithelial cell‐deposited Cr(VI) is reduced to trivalent chromium [Cr(III)] by glutathione, resulting in large amounts of reactive oxygen species (ROS).^4^ Because Cr(III) cannot cross cell membranes, once inside a cell, it is trapped and crosslinked to chromatin and organelles; this ultimately causes genotoxicity and epigenetic dysregulation.^4^

Aldehyde dehydrogenase 1A1 (ALDH1A1) links aldehydes to carboxylic acids through NAD(P)^+^‐dependent oxidation,^5^ and therefore plays critical roles in cellular detoxication and ROS scavenging. ALDH1A1 is a marker of cancer stem cells (CSCs),^6^ which play vital roles in self‐renewal, differentiation, and self‐protection of several types of cancers, including lung,^7^ liver,^8^ ovarian,^9^ pancreatic,^10^ and breast cancers.^11^, ^12^ ALDH1A1 affects CSCs mainly through its metabolic product, retinoic acid (RA), which is the ligand for the RA receptor (RAR), a nuclear receptor that functions as a transcription factor to regulate the downstream target gene expressions.^13^ Without its ligand, RAR interacts with retinoid X receptor (RXR) and forms heterodimers recruiting corepressors to chromatin for the maintenance of cellular transcriptional inactivity.^14^ Once RA binds, the RAR/RXR dimer detaches from corepressors and subsequently recruits coactivators to RA response elements (RAREs) on the genome, which in turn promote the transcriptional activity of downstream target genes.^14^

Here, we report that ALDH1A1 expression is induced during Cr(VI)‐mediated bronchial epithelial cell malignant transformation. ALDH1A1 drives the maintenance of a CSC‐like subgroup of lung cancer cells and transcriptionally enhances EGF expression, promoting LUSC tumourigenesis.

## 2 RESULTS

### 2.1 Cr(VI) exposure increases ALDH1A1 expression in transformed bronchial epithelial cells

Chronic exposure to Cr(VI) can result in lung carcinoma.^15^ To investigate the potential mechanism, we established an *in* *vitro* malignant transformed cell model by exposing BEAS‐2B cells to various amounts of K_2_Cr_2_O_7_ (Figure S1A). After exposure for 18 months, the colony formation rate and the xenograft tumour formation rate of Cr(VI)‐treated BEAS‐2B cells were > 80%, indicating that Cr(VI)‐transformed cell line (hereafter termed CrT) was established successfully (Figure S1A). The tolerance of CrT cells to Cr(VI) treatment was much higher than that of BEAS‐2B cells (Figure S1B). Importantly, the malignancy of CrT cells was inheritable as evidenced by the colony formation rate and xenograft tumour formation rate of CrT cells passaged more than 30 generations, which were both still > 80% (Figure S1C–E). All subsequent experiments were performed with CrT cells passaged less than 30 generations.

**FIGURE 1:**
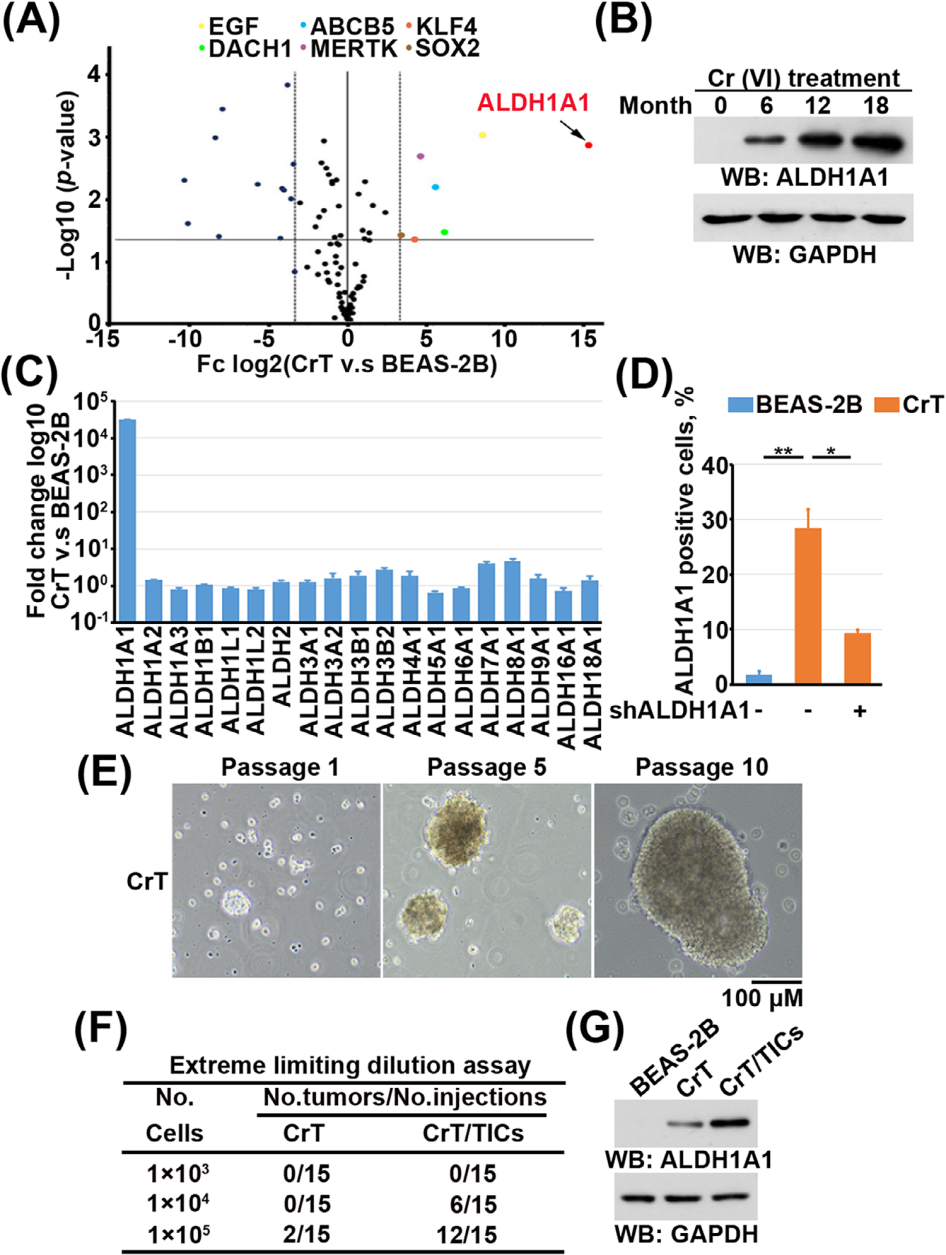
Cr(VI) exposure induces ALDH1A1 expression and stemness characteristics. (A) Volcano map of the gene arrays. The horizontal axis corresponds to 10‐fold upregulation, and the vertical axis represents the *P*‐value. (B) BEAS‐2B cells exposed to Cr(VI) for the indicated periods were lysed for immunoblot analysis. (C) Quantitative Real‐Time PCR (qRT‐PCR) analysis of ALDH family gene mRNA levels. Data are presented as the mean ± SD of triplicate experiments. (D) BEAS‐2B or CrT cells with or without ALDH1A1 depletion were used for the detection of ALDH1A1 activity by flow cytometry. Data are presented as the mean ± SD of triplicate experiments. *P < .01, **P < .001. (E) Representative phase‐contrast images of tumourspheres derived from CrT cells at the indicated passages. Scale bar, 100 μm. (F) Frequency of lung orthotopic tumourigenesis after injection of the indicated amounts of CrT and CrT/TICs. (G) BEAS‐2B cells, CrT cells, and CrT/TICs were lysed for immunoblot analysis with the indicated antibodies.

Cr(VI) exposure has been reported to induce CSC‐like properties.^16^ Analysis of the differentially expressed CSC markers revealed that ALDH1A1 was the most strongly upregulated CSC marker in CrT cells compared with BEAS‐2B cells (Figure 1A, Table S1). The protein levels of ALDH1A1 were progressively upregulated during the Cr(VI)‐induced transformation process (Figure 1B). In addition, among all 19 ALDH superfamily members, ALDH1A1 was the only one that was upregulated in CrT cells compared with BEAS‐2B cells (Figure 1C). Flow cytometry analysis revealed that ∼28% of CrT cells were ALDH1A1‐positive (Figures 1D and S1F), suggesting the existence of a CSC‐like subpopulation within CrT cells. This subpopulation, hereafter termed tumour‐initiating cells (CrT/TICs), was selected and enriched by 3D culture using serum‐free 3dGRO Spheroid Medium. The CSC property of CrT/TICs was validated by the exhibition of sphere‐like growth (Figure 1E); moreover, in an *in* *vivo* extreme limiting dilution assay, the isolated CrT/TICs demonstrated a much stronger tumour formation ability than the parental CrT cells (Figures 1F and S1G). Finally, the protein levels of ALDH1A1 were much increased in CrT/TICs compared to CrT cells (Figure 1G). Collectively, these results suggest that Cr(VI) exposure induces ALDH1A1 expression and CSCs subpopulation formation.

### 2.2 Cr(VI)‐induced overexpression of ALDH1A1 maintains self‐renewal of CrT/TICs

**FIGURE 2:**
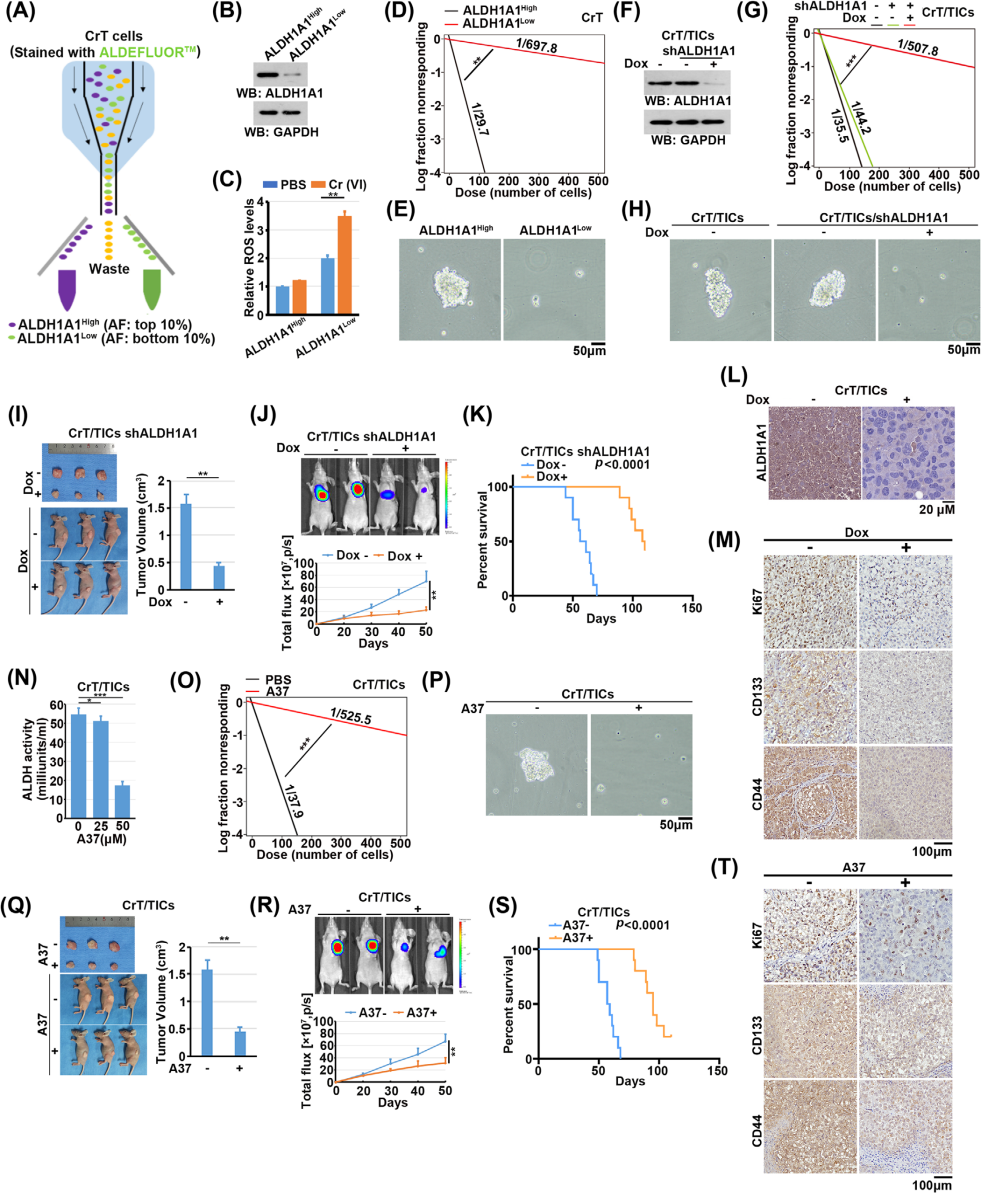
Cr(VI)‐induced overexpression of ALDH1A1 maintains self‐renewal of CrT/TICs. (A) Cell sorting for ALDH1A1^High^ and ALDH1A1^Low^ CrT cells. CrT cells were stained with ALDEFLUOR kit and PI. ALDH1A1^High^ cells: AF top 10%, PI (–); ALDH1A1^Low^ cells: AF bottom 10%, PI (–). AF: ALDEFLUOR Fluorescence. (B) ALDH1A1^High^ and ALDH1A1^Low^ CrT cells were lysed for immunoblot analyses with the indicated antibodies. (C) Reactive oxygen species (ROS) levels were detected by DCFH‐DA staining in ALDH1A1^Low^ and ALDH1A1^High^ CrT cells exposed with or without Cr (VI). Data represent the mean ± SD of triplicate experiments. **p  < .001. (D) *In* *vitro* limiting dilution assays on ALDH1A1^High^ and ALDH1A1^Low^ CrT cells. **p < .001. (E) Tumoursphere formation assays using ALDH1A1^High^ and ALDH1A1^Low^ CrT cells. (F) CrT/TICs with or without Dox‐inducible *ALDH1A1* shRNA were treated with or without Dox and lysed for immunoblot analyses with the indicated antibodies. (G) *In* *vitro* limiting dilution assays on CrT/TICs cells with or without doxycycline (Dox)‐inducible *ALDH1A1* shRNA. **p < .001. (H) Tumoursphere formation assays using CrT/TICs with or without Dox‐inducible *ALDH1A1* shRNA. (I) CrT/TICs with Dox‐inducible *ALDH1A1* shRNA were subcutaneously implanted in the left side of mice. (J) CrT/TICs with Dox‐inducible *ALDH1A1* shRNA were orthotopically implanted in the lung of mice. (Top) Representative BLIs of lung orthotopic tumours with or without Dox treatment for 50 days. (Bottom) Quantification of BLIs every 10 days. Data are presented as the mean ± SD from five mice. **P < .001. (K) Kaplan–Meier survival curves for indicated mice. (L) Immunohistochemical (IHC) staining was performed with antibody against ALDH1A1. Scale bar, 20 μm. (M) IHC staining was performed with antibodies against Ki‐67, CD133, and CD44. Scale bar, 20 μm. (N) ALDH1A1 activity were detected in CrT/TICs with the indicated concentration of A37. Data represent the mean ± SD of triplicate experiments. *p < .01, ***p < .0001. (O) *In* *vitro* limiting dilution assays on CrT cells treated with or without A37 (50 μM). ***p < .0001. (P) Tumoursphere formation assays using CrT cells treated with or without A37 (50 μM). (Q) CrT/TICs were subcutaneously implanted in the left side of mice. (R) CrT/TICs were orthotopically implanted in the lung of mice. (Top) Representative BLIs of lung orthotopic tumours with or without A37 treatment for 50 days. (Bottom) Quantification of BLIs every 10 days. Data are presented as the mean ± SD from five mice. **P < .001. (S) Kaplan–Meier survival curves for indicated mice. (T) IHC staining was performed with antibodies against Ki‐67, CD133, and CD44. Scale bar, 20 μm

To test the effects of ALDH1A1 expression on CrT cells, we sorted the top 10% and bottom 10% of CrT cells in regard to the ALDH1A1 activity; these cells were designated as ALDH1A1^High^ and ALDH1A1^Low^, respectively (Figure 2A,B). In agreement with the observation that ALDH1A1 acts as a ROS scavenger,^17^ ALDH1A1^High^ CrT cells exhibited a stronger ability to eliminate Cr(VI)‐induced ROS than ALDH1A1^Low^ cells (Figure 2C). An *in* *vitro* limiting dilution coupled with sphere formation assay demonstrated that ALDH1A1^High^ CrT cells displayed a stronger self‐renewal capacity than ALDH1A1^Low^ CrT cells in the serum‐free 3D tumoursphere suspension culture system (Figure 2D,E).

To test the effects of ALDH1A1 on CrT/TICs, we used doxycycline‐inducible ALDH1A1 depletion system. Doxycycline treatment successfully induced ALDH1A1 depletion (Figure 2F) and dramatically reduced the self‐renewal capacity of CrT/TICs (Figure 2G,H). To detect the role of ALDH1A1 *in* *vivo*, CrT/TICs were subcutaneously and left lung‐orthotopically implanted into immunodeficient mice. Doxycycline administration inhibited tumour growth and prolonged the survival of mice (Figure 2I–K). Immunohistochemical staining confirmed the successful depletion of ALDH1A1 in xenograft tissues (Figure 2L). Moreover, the cell proliferation marker Ki‐67 and stem cell markers including CD133 and CD44 were decreased in the ALDH1A1 depletion group (Figure 2 M). We next tested the effects of the selective small‐molecule ALDH1A1 inhibitor A37 on CrT/TICs.^18^ As expected, A37 treatment decreased ALDH1A1 activity (Figure 2N), as well as the self‐renewal capacity of CrT/TICs (Figure 2O,P). In line with ALDH1A1 depletion, A37 largely impaired tumour growth (Figure 2Q,R) and prolonged survival of mice bearing CrT/TICs (Figure 2S). In addition, in CrT/TIC xenografts, Ki‐67, CD133, and CD44 levels were decreased in response to A37 treatment (Figure 2T). The above data conclude that ALDH1A1 plays essential roles in the maintenance of CSC features of Cr(VI)‐transformed cells.

### 2.3 Cr(VI) induces ALDH1A1 expression through KLF4

**FIGURE 3:**
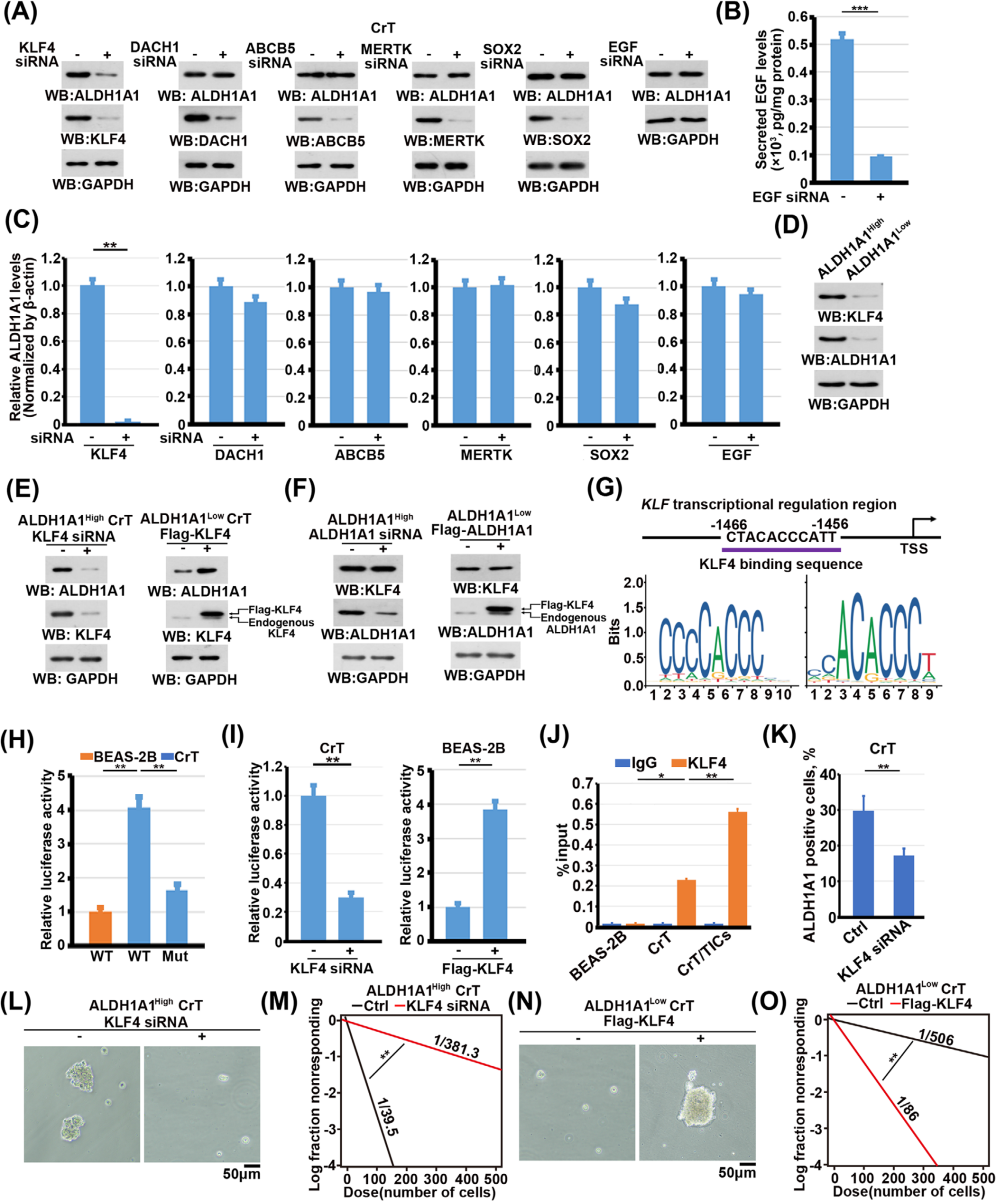
Cr(VI) induces ALDH1A1 expression through KLF4. (A) CrT cells transfected with siRNAs (50 nM) targeting *KLF4, DACH1, ABCB5, MERTK, SOX2* or *EGF* for 72 h and were lysed for immunoblot analyses with the indicated antibodies. (B) CrT cells transfected with or without *EGF* siRNA (50 nM, 72 h) were lysed for ELISA analyses for detecting secreted EGF levels in the culturing media. (C) CrT cells transfected with siRNAs (50 nM) targeting *KLF4, DACH1, ABCB5, MERTK, SOX2* or *EGF* for 72 h and were lysed for qRT‐PCR analysis of *ALDH1A1* mRNA expression levels. Data are presented as the mean ± SD of triplicate experiments. **P < .001. (D) ALDH1A1^High^ and ALDH1A1^Low^ CrT cells were lysed for immunoblot analyses with the indicated antibodies. (E) ALDH1A1^Low^ CrT cells transfected with or without Flag‐KLF4 for 72 h were lysed for immunoblot analysis with the indicated antibodies. ALDH1A1^Low^ CrT cells transfected with or without Flag‐ALDH1A1 were lysed for immunoblot analyses with the indicated antibodies. (F) ALDH1A1^Low^ CrT cells transfected with or without *KLF4* siRNA (50 nM) for 72 h were lysed for immunoblot analysis with the indicated antibodies. ALDH1A1^High^ CrT cells transfected with or without *ALDH1A1* siRNA were lysed for immunoblot analyses with the indicated antibodies. (G) Schematic image represents the KLF4 binding sequence within the *ALDH1A1* transcriptional regulation region. (H) Luciferase reporter assays were performed in BEAS‐2B and CrT cells transfected with pGL‐3.0 vector containing *ALDH1A1* WT or mutant promoter. Data represent the mean ± SD of triplicate experiments. **p < .001. (I) CrT cells with or without KLF4 depletion and BEAS‐2B cells with or without expression of Flag‐KLF4 were transfected with a luciferase reporter gene under the control of the *ALDH1A1* promoter for 24 h. Luciferase reporter assays were performed. Data are presented as the mean ± SD of triplicate experiments. **P < .001. (J) BEAS‐2B cells, CrT cells, and CrT/TICs were used for ChIP‐qPCR analysis of the *ALDH1A1* promoter with the indicated antibody. Data are presented as the mean ± SD of triplicate experiments. *P < .01, **P < .001. (K) CrT cells with or without KLF4 depletion were used for the detection of ALDH1A1 activity by flow cytometry. Data are presented as the mean ± SD of triplicate experiments. **P < .001. (L) Tumoursphere formation assays using ALDH1A1^High^ CrT cells transfected with or without *KLF4* siRNA. (M) *In* *vitro* limiting dilution assays on ALDH1A1^High^ CrT cells transfected with or without *KLF4* siRNA. **p < .001. (N) *In* *vitro* limiting dilution assays on ALDH1A1^Low^ CrT cells transfected with or without Flag‐KLF4. **p < .001. (O) Tumoursphere formation assays using ALDH1A1^Low^ CrT cells transfected with or without Flag‐KLF4

To understand how ALDH1A1 was regulated by Cr(VI), we examined the top‐upregulated genes in CrT cells compared with BEAS‐2B cells (Figure 1A). Suppression of *KLF4*, but not other genes, including *DACH1*, *ABCB5*, *MERTK*, *SOX2*, and *EGF*, decreased both protein and mRNA levels of ALDH1A1 in CrT cells (Figure 3A–C). The expression level of KLF4 was much lower in ALDH1A1^low^ CrT cells than in ALDH1A1^High^ cells (Figure 3D), suggesting a positive correlation between ALDH1A1 and KLF4. Overexpression and depletion of KLF4 upregulated and suppressed ALDH1A1 expression in CrT cells (Figure 3E), respectively. However, neither overexpression nor depletion of ALDH1A1 regulated expression KLF4 (Figure 3F), suggesting that KLF4 acts as an upstream regulator of ALDH1A1. KLF4 is a Yamanaka transcription factor that orchestrates various cellular processes.^19^, ^20^ The region from −1466 to −1456 upstream of the transcription start site of *ALDH1A1* was predicted to be a putative binding site of KLF4 (Figure 3G). CrT cells containing the putative KLF4 binding region of the *ALDH1A1* promoter exhibited high luciferase reporter activity compared with BEAS‐2B cells; this effect was abrogated when the putative region was mutated (Figure 3H). Furthermore, overexpression of KLF4 in BEAS‐2B cells enhanced and depletion of KLF4 in CrT cells decreased the activity of this luciferase reporter (Figure 3I). Chromatin immunoprecipitation (ChIP) analysis revealed that KLF4 was strongly enriched at the *ALDH1A1* promoter region in both CrT cells and CrT/TICs (Figure 3J), suggesting that KLF4 is a potential transcriptional factor controlling *ALDH1A1* expression. Consistently, KLF4 depletion strongly reduced the proportion of ALDH1A1‐positive CrT cells (Figure 3K). An *in* *vitro* limiting dilution coupled with sphere formation assay demonstrated that KLF4 depletion impaired the self‐renewal capacity of ALDH1A1^High^ CrT cells (Figure 3L,M), while KLF4 overexpression enhanced this ability in ALDH1A1^low^ CrT cells (Figure 3N,O). These results demonstrate that KLF4 activates *ALDH1A1* transcription and maintains the CSC‐like properties of CrT cells.

### 2.4 ALDH1A1 promotes EGF expression in CrT/TICs

**FIGURE 4:**
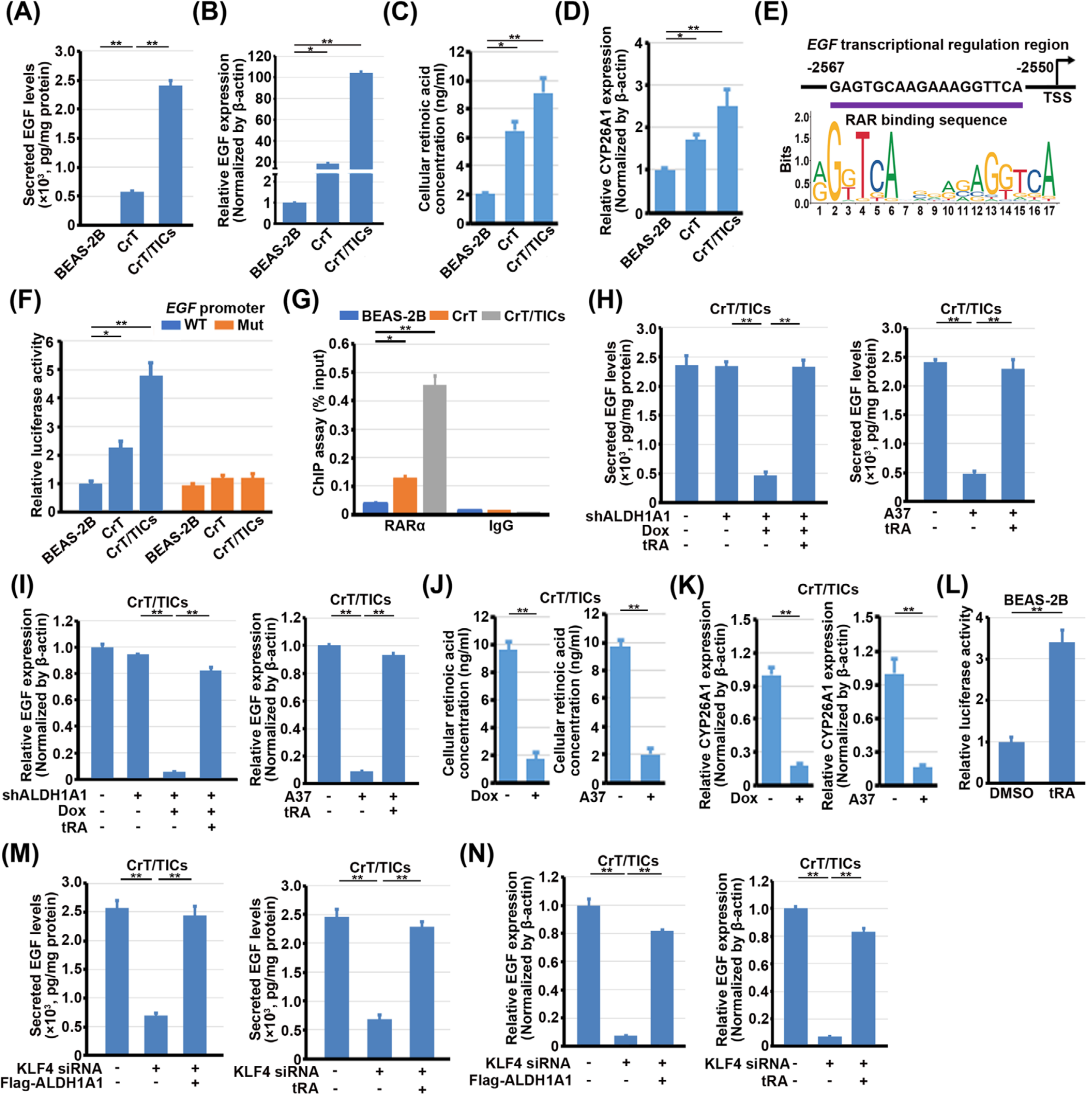
ALDH1A1 promotes EGF expression in CrT/TICs. (A) ELISA analysis of secreted EGF levels in the culture media of BEAS‐2B cells, CrT cells, and CrT/TICs. Data are presented as the mean ± SD of triplicate experiments. **P < .001. (B) qRT‐PCR analysis of EGF mRNA levels in BEAS‐2B cells, CrT cells and CrT/TICs. Data represent the mean ± SD of triplicate experiments. *p < .01, **p < .001. (C) Cellular retinoic acid levels were tested in indicated cells. *p < .05. **p < .001. (D) CYP26A1 levels were tested in indicated cells. *p < .05. **p < .001. (E) Schematic image represents the RARα binding sequence within the *EGF* transcriptional regulation region. (F) The luciferase reporter gene under the control of the wild‐type (WT) or mutant (Mut) *EGF* promoter was transfected in the indicated cells for 24 h. Luciferase reporter assays were performed. Data are presented as the mean ± SD of triplicate experiments. *P < .05. **P < .001. (G) The indicated cells were used for ChIP‐qPCR analysis of the *EGF* promoter with the indicated antibody. Data are presented as the mean ± SD of triplicate experiments. *P < .05. **P < .001. (H) CrT/TICs with or without Dox (100 ng/ml, 72 h) and A37 treatment (50 μM) in the presence or absence of tRA (1 μM). The secreted levels of EGF were measured by ELISA. Data are presented as the mean ± SD of triplicate experiments. **P < .001. (I) CrT/TICs with or without Dox (100 ng/ml) and A37 treatment (50 μM) in the presence or absence of tRA (1 μM). EGF mRNA levels were measured by qRT‐PCR analyses. Data represent the mean ± SD of triplicate experiments. **p < .001. (J) Cellular retinoic acid levels were tested in indicated cells. **p < .001. (K) CYP26A1 levels were tested in indicated cells. **p < .001. (L) BEAS‐2B cells transfected with the luciferase reporter gene under the control of the *EGF* promoter were treated with or without tRA (1 μM) for 24 h. The luciferase reporter assay was performed. Data are presented as the mean ± SD of triplicate experiments. **P < .001. (M) KLF4‐depleted CrT/TICs were transfected with or without Flag‐ALDH1A1 or treated with or without tRA (1 μM). The secreted levels of EGF were measured by ELISA. Data are presented as the mean ± SD of triplicate experiments. **P < .001. (N) KLF4‐depleted CrT/TICs were transfected with or without Flag‐ALDH1A1 or treated with or without tRA (1 μM). EGF mRNA levels were measured by qRT‐PCR analyses. Data represent the mean ± SD of triplicate experiments. **p < .001.

EGF, the ligand of EGFR, was one of the most strongly upregulated genes among Cr(VI)‐induced genes (Figure 1A). Because Cr(VI) exposure activates the EGFR signal pathway,^21^, ^22^ but the underlying mechanism remains undetermined, we speculated that Cr(VI) induces EGF expression through ALDH1A1. Both mRNA levels and secreted protein levels of EGF were upregulated in CrT cells, especially in the CrT/TIC subgroup (Figure 4A,B). ALDH1A1 exerts transcriptional regulation through its metabolic product, RA, as well as RAR.^13^, ^23^ Additionally, RA levels in CrT cells were increased, especially in the CrT/TIC subgroup (Figure 4C). The expression levels of *CYP26A1*, which immediately responds to the RA concentration, were also increased in CrT cells, especially in the CrT/TIC subgroup (Figure 4D). The putative RAR binding region within the *EGF* promoter was predicted to be located from −2567 to −2550 upstream of the transcription start site (Figure 4E). In agreement with the expression levels of EGF, the luciferase reporter containing this region of the *EGF* promoter was shown to be largely activated in CrT and CrT/TICs; this activation was abrogated when this putative region was mutated (Figure 4F). ChIP assays validated that RAR is directly bound to the *EGF* promoter (Figure 4G). Both doxycycline‐induced depletion and A37‐mediated enzymatic inhibition of ALDH1A1 resulted in decreased mRNA levels and secreted protein levels of EGF in CrT/TICs, accompanied by decreased levels of RA and *CYP26A1* (Figure 4H–K). Treatment with all‐trans RA (tRA), which activates RAR, activated the *EGF* promoter in BEAS‐2B cells (Figure 4L) and restored EGF expression levels in CrT/TICs, where ALDH1A1 was suppressed (Figure 4H,I). Because no putative KLF4 binding region was found in the *EGF* promoter, we examined whether KLF4 regulates EGF expression through ALDH1A1. Depletion of KLF4 downregulated both mRNA levels and secreted protein levels of EGF in CrT/TICs (Figure 4M,N). This downregulation was restored by overexpression of ALDH1A1 or treatment with tRA in CrT/TICs (Figure 4M,N). These data show Cr(VI)‐induced ALDH1A1 promotes EGF expression in CrT/TICs.

### 2.5 CrT/TIC‐secreted EGF activates EGFR signalling and promotes lung cancer cell proliferation

**FIGURE 5:**
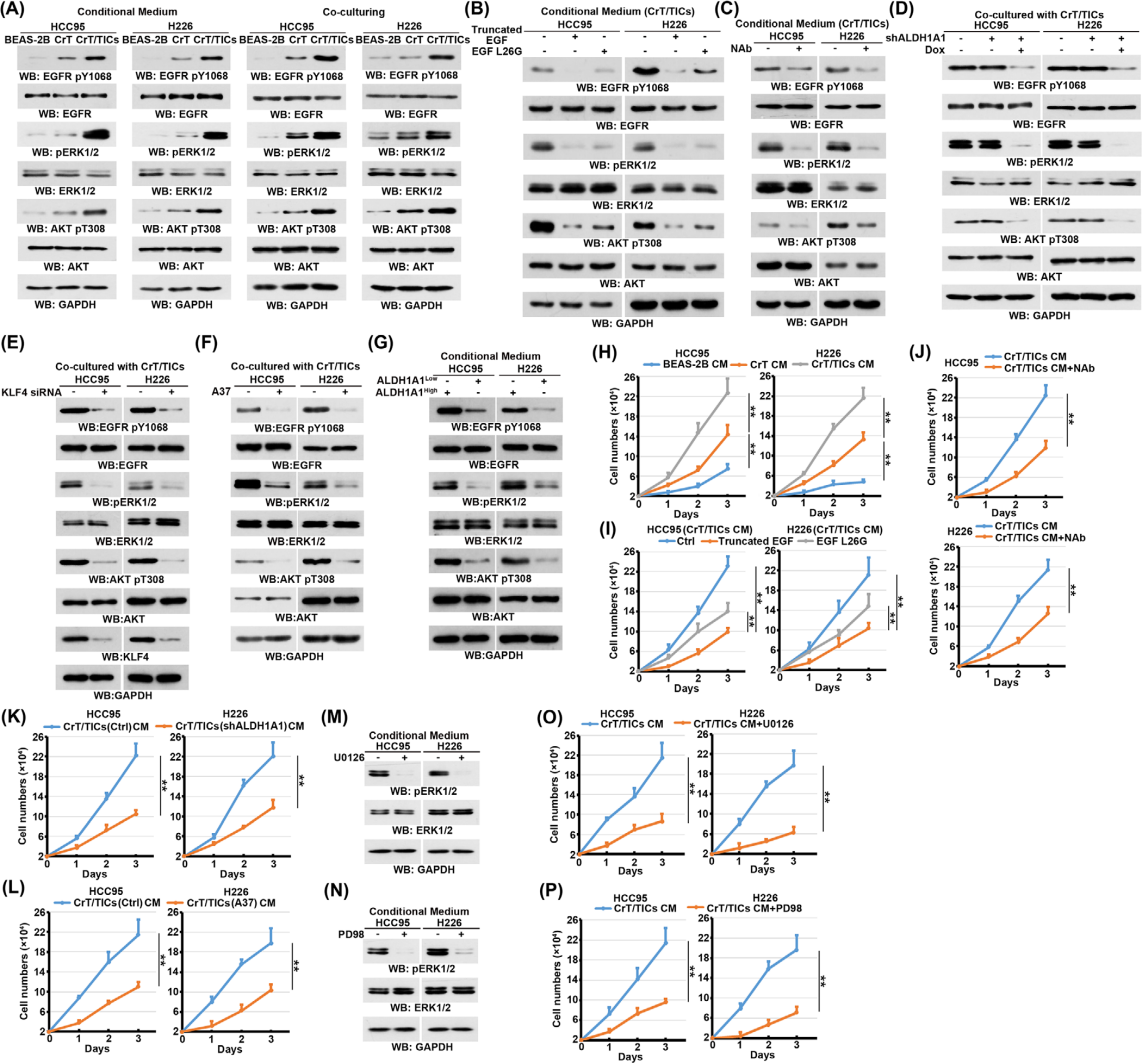
CrT/TIC‐secreted EGF activates EGFR signalling and promotes LUSC cell growth. (A) HCC95 and H226 cells incubated with a conditioned medium or co‐cultured with the indicated cells were lysed for immunoblot analysis with the indicated antibodies; HCC95 and H226 cells co‐cultured with CrT/TICs transfected with or without *KLF4* siRNA were lysed for immunoblot analyses with the indicated antibodies. (B) HCC95 and H226 cells incubated with CrT/TIC‐derived conditioned medium for 12 h in the presence or the absence of human recombinant truncated EGF or EGF L26G were lysed for immunoblot analysis with the indicated antibodies; HCC95 and H226 cells co‐cultured with CrT/TICs transfected with or without KLF4 siRNA were lysed for immunoblot analyses with the indicated antibodies. (C) HCC95 and H226 cells incubated with CrT/TIC‐derived conditioned medium for 12 h in the presence or the absence of EGF‐neutralising antibodies were lysed for immunoblot analyses with the indicated antibodies. (D) HCC95 and H226 cells co‐cultured with CrT/TICs with or without ALDH1A1 depletion were lysed for immunoblot analyses with the indicated antibodies. (E) HCC95 and H226 cells co‐cultured with CrT/TICs transfected with or without KLF4 siRNA were lysed for immunoblot analyses with the indicated antibodies. (F) HCC95 and H226 cells co‐cultured with CrT/TICs pretreated with or without A37 were lysed for immunoblot analyses with the indicated antibodies. (G) HCC95 and H226 cells incubated with conditional medium derived from ALDH1A1^Low^ CrT or ALDH1A1^High^ CrT were lysed for immunoblot analyses with the indicated antibodies. (H) Growth curves of HCC95 and H226 cells cultured with BEAS‐2B‐, CrT‐, and CrT/TIC‐derived conditioned medium. Data are presented as the mean ± SD of triplicate experiments. **P < .001. (I) Growth curves of HCC95 and H226 cells cultured with CrT/TIC‐derived conditioned medium pretreated with truncated EGF or EGF L26G. Data are presented as the mean ± SD of triplicate experiments. **P < .001. (J) Growth curves for the HCC95 and H226 cells cultured with CrT/TICs‐derived conditional medium pretreated with or without anti‐EGF antibody. Data represent the mean ± SD of triplicate experiments. **p < .001. (K) Growth curves of HCC95 and H226 cells cultured with the indicated conditioned medium derived from CrT/TICs with or without ALDH1A1 depletion. Data are presented as the mean ± SD of triplicate experiments. **P < .001. (L) Growth curves of HCC95 and H226 cells cultured with the indicated conditioned medium derived from CrT/TICs with or without A37 treatment. Data are presented as the mean ± SD of triplicate experiments. **P < .001. (M) HCC95 and H226 cells co‐cultured with CrT/TICs pretreated with or without U0126 were lysed for immunoblot analyses with the indicated antibodies. (N) HCC95 and H226 cells co‐cultured with CrT/TICs pretreated with or without PD98 were lysed for immunoblot analyses with the indicated antibodies. (O) Growth curves of HCC95 and H226 cells with or without U0126 treatment cultured with the indicated conditioned medium derived from CrT/TICs. Data are presented as the mean ± SD of triplicate experiments. **P < .001. (P) Growth curves of HCC95 and H226 cells with or without PD98 treatment cultured with the indicated conditioned medium derived from CrT/TICs. Data are presented as the mean ± SD of triplicate experiments. **P < .001.

The crosstalk between CSCs and differentiated tumour cells contributes to tumourigenesis, metastasis, recurrence, and therapeutic resistance.^24^ We therefore tested whether CrT/TICs secrete EGF to activate EGFR signalling in differentiated lung cancer cells. To test this, we co‐cultured HCC95 and H226 human LUSC cells with CrT/TICs, CrT cells, and BEAS‐2B cells. Co‐culturing with CrT/TICs or with their derived conditioned medium resulted in the strongest EGFR signalling activation, as evidenced by phosphorylation of EGFR, ERK1/2, and AKT in LUSC cells, compared with CrT or BEAS‐2B cells (Figure 5A). This activation was abrogated by neutralization of EGF in CrT/TIC‐derived conditioned medium through truncated EGF and EGF L26G, an inactive EGF mutant (Figure 5B), or with anti‐EGF antibody (Figure 5C). Inhibition of ALDH1A1 in CrT/TICs by doxycycline‐induced ALDH1A1 depletion, with the ALDH1A1 inhibitor A37, or by siRNA‐mediated KLF4 depletion suppressed CrT/TIC‐activated EGFR signalling in HCC95 and H226 cells (Figure 5D–F). Consistently, the conditioned medium from ALDH1A1^High^ CrT cells strongly activated EGFR signalling in HCC95 and H226 cells compared with ALDH1A1^low^ cells (Figure 5G).

The strongest proliferation capacity of HCC95 and H226 cells was observed when cultured in a conditioned medium from CrT/TICs, compared with a conditioned medium from CrT and BEAS‐2B cells (Figure 5H). Neutralisation of EGF by truncated EGF, EGF L26G, or anti‐EGF antibody in the CrT/TIC‐derived conditioned medium suppressed the proliferation of HCC95 and H226 cells (Figure 5I,J). In line with these results, inhibition of ALDH1A1 in CrT/TICs through doxycycline‐induced depletion or by A37 treatment largely abrogated the effects of CrT/TIC‐derived conditioned medium on the proliferation of HCC95 and H226 cells (Figure 5K,L). Further, as EGFR acts upstream of the ERK signal pathway, we also treated the cells with the ERK inhibitors U0126 and PD98. Both U0126 and PD98 exerted significant inhibitory effects on HCC95 and H226 cells (Figure 5M,N). As expected, ERK inhibition in HCC95 and H226 cells largely abrogated the stimulatory effects of CrT/TIC‐derived conditioned medium on proliferation (Figure 5O,P). These results suggest that CrT/TIC‐secreted EGF activates EGFR and promotes LUSC cell proliferation through Cr(VI)‐induced ALDH1A1 expression.

### 2.6 CrT/TICs support tumour growth of differentiated LUSC cells, and ALDH1A1 inhibition abrogates these effects

**FIGURE 6:**
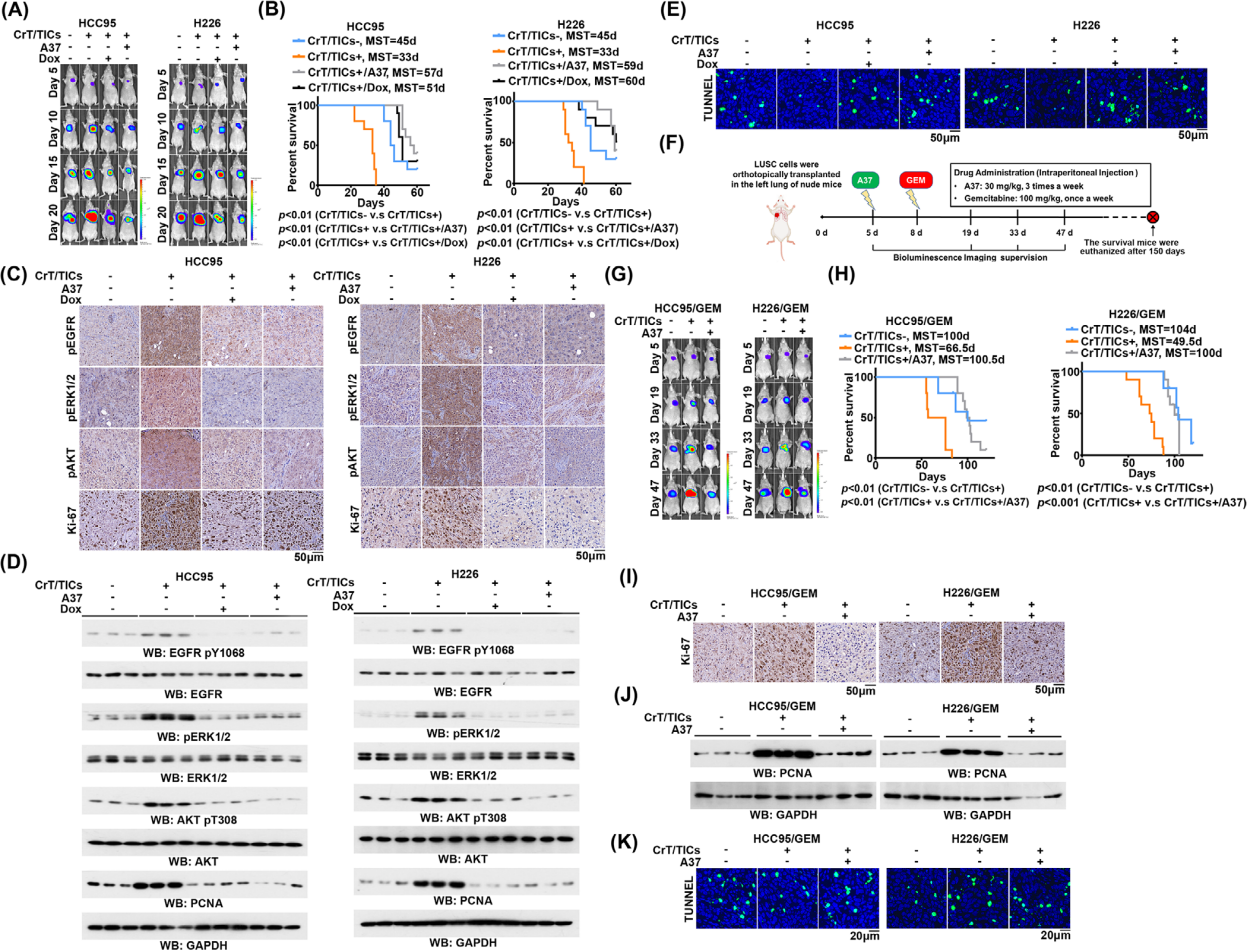
ALDH1A1 inhibition increases the anti‐tumour effects of gemcitabine. (A) HCC95 or H226 cells (2 × 10^6^) mixed with or without CrT/TICs (1 × 10^3^) that stably expressed Dox‐inducible *ALDH1A1* shRNA were orthotopically transplanted in the lungs of mice. After 5 days, mice were intraperitoneally injected with A37 or Dox triplicate times per week. Representative BLIs of orthotopic tumours and quantification of BLIs every 5 days are shown. (B) Kaplan–Meier survival curves for indicated mice. (C) IHC staining was performed with the indicated antibodies. Scale bar, 50 μm. (D) Tumour tissues were collected and homogenate. Immunoblot was conducted with indicated antibodies. (E) Representative TUNEL staining (green) and corresponding DAPI nuclear staining (blue) for indicated cells. (F) Drug treatment regimen for administration of A37 and gemcitabine (GEM). (G) HCC95 or H226 cells (2 × 10^6^) mixed with or without CrT/TICs (1 × 10^3^) were orthotopically transplanted in the lungs of mice. After 5 days, mice were treated with GEM. Representative BLIs of orthotopic tumours and quantification of BLIs every 14 days are shown. (H) Kaplan–Meier survival curves for indicated mice. MST, median survival time. (I) IHC staining was performed with anti‐Ki‐67. Scale bar, 50 μm. (J) Tumour tissues were collected and homogenate. Immunoblot was conducted with indicated antibodies. (K) Representative TUNEL staining (green) and corresponding DAPI nuclear staining (blue) for indicated cells

To determine whether CrT/TICs contribute to the tumourigenesis of differentiated lung cancer cells, we orthotopically implanted luciferase‐expressing HCC95 or H226 cells with or without the certain types of CrT/TICs into the lungs of nude mice. Mixing with CrT/TICs tremendously accelerated lung tumour growth of HCC95 or H226 cells (Figure 6A) and reduced the survival duration of mice (Figure 6B). In line with their ability to promote tumour growth, mixing with CrT/TICs strongly enhanced EGFR signalling activation, as demonstrated by the increased phosphorylation levels of EGFR, ERK1/2, and AKT, increased the proliferation ability, as demonstrated by increased levels of Ki‐67 and PCNA (Figure 6C,D), and decreased apoptosis in xenograft tumours (Figures 6E). To test whether these effects were dependent on elevated expression of ALDH1A1 in CrT/TICs, we treated mice with doxycycline or A37 to inhibit ALDH1A1. Doxycycline or A37 treatment decreased the CrT/TIC mixing‐induced tumour growth of HCC95 and H226 cells (Figure 6A) and prolonged the survival of mice (Figure 6A). Consistently, both doxycycline and A37 decreased the activity of EGFR signalling and the levels of proliferation markers (Figure 6C,D) and enhanced apoptosis of xenograft tumours (Figure 6E).

CSCs also contribute to the acquired resistance of cancer cells to chemotherapeutic agents.^25^, ^26^ We therefore tested the effects of the selective ALDH1A1 inhibitor A37 combined with gemcitabine on lung tumour growth (Figure 6F). Resistance to gemcitabine was observed in xenografts derived from HCC95 and H226 cells when they were mixed with CrT/TICs; this resistance was strongly alleviated by co‐administration of A37 (Figure 6G). In agreement with these observations, combined treatment greatly prolonged the survival of mice bearing lung tumours (Figure 6H), reduced Ki‐67 and PCNA levels (Figure 6I and J), and increased the proportion of apoptotic cells in these xenografts (Figure 6K) compared with gemcitabine treatment alone. These results suggest that the inhibition of ALDH1A1 may serve as an adjuvant therapy to improve the efficiency of gemcitabine.

### 2.7 ALDH1A1 is positively correlated with EGFR signalling and predicts poor prognosis of LUSC patients

**FIGURE 7:**
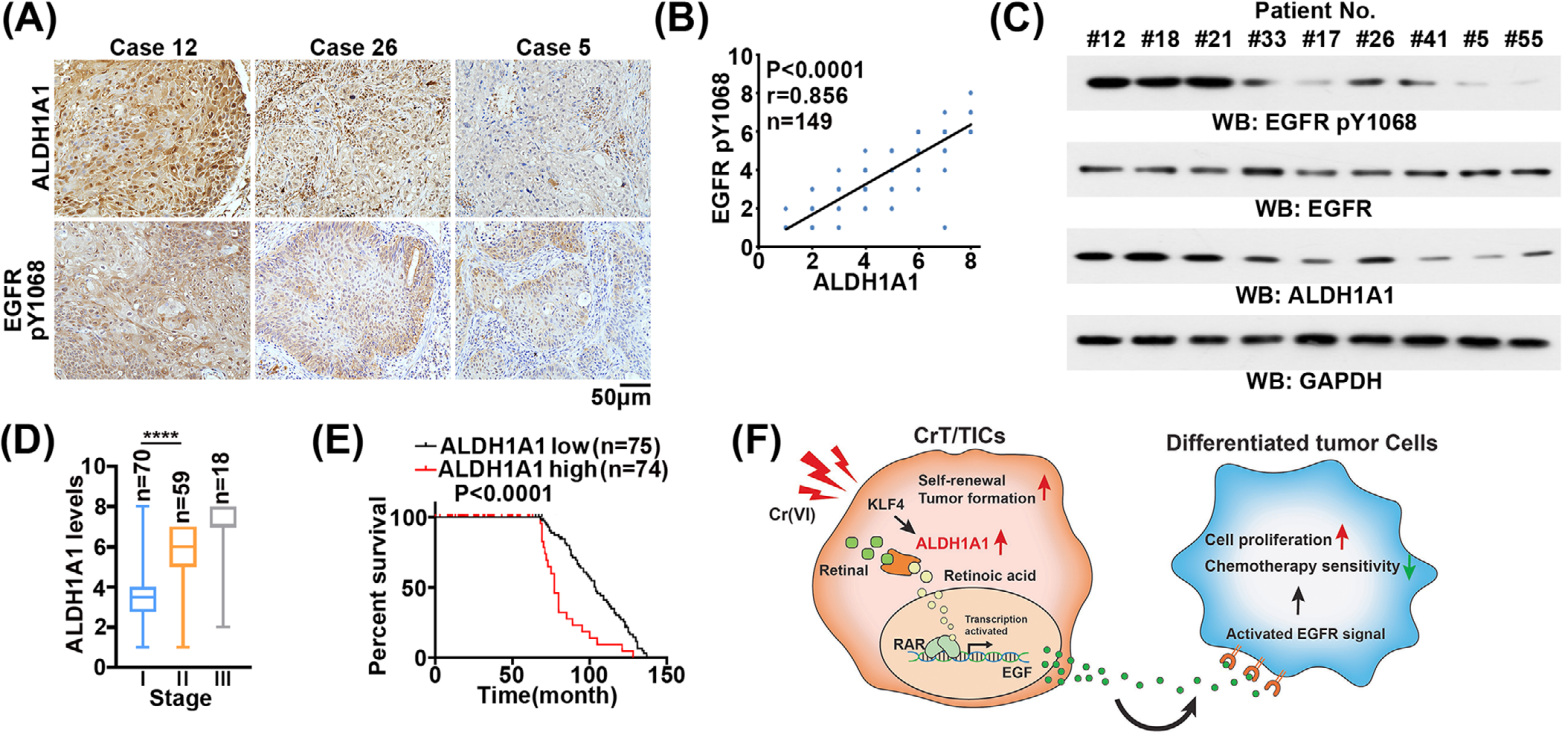
ALDH1A1 expression is positively correlated with EGFR signalling and predicts poor prognosis. (A) IHC staining was performed with the indicated antibodies. Scale bar, 50 μm. (B) Correlation between ALDH1A1 levels and EGFR pY1068 levels in LUSC samples. Spearman's correlation tests were performed. Note that some of the dots represent more than one specimen. (C) The protein was extracted from patients’ paraffin sections to conduct immunoblot analysis with the indicated antibodies. (D) Box plot shows the ALDH1A1 expression in Stages I, II, and III of LUSC patients. ****P < .00001. (E) Kaplan–Meier survival curves for LUSC patients, ALDH1A1 high staining vs. ALDH1A1 low staining. (F) Cr (VI) exposure induces malignant transformation of bronchial epithelial cells (CrT), which contain a subpopulation of cancer stem cell (CSC)/tumour initiating cell (TIC)‐like cells (CrT/TICs). These cells are characterised with high expression of ALDH1A1, which is transcriptionally activated by KLF4 in response to Cr (VI) challenges. The highly expressed ALDH1A1 maintains self‐renewal capacity of CrT/TICs and promotes EGF secretion from CrT/TICs in a transcriptional‐dependent manner. The secreted EGF subsequently activates EGFR signalling in the differentiated tumour cells and promotes tumourigenesis of LUSC.

To determine the correlation between ALDH1A1 levels and EGFR activation, 149 human LUSC samples were collected for IHC staining using antibodies against ALDH1A1 and EGFR pY1068 (Figure 7A). The quantitative scores of IHC staining and immunoblot data shown ALDH1A1 were positively correlated with EGFR pY1068 (Figure 7B,C). To clarify the clinical significance of ALDH1A1, the IHC staining scores of ALDH1A1 in LUSC samples at different clinical stages were analysed. Expression levels of ALDH1A1 were found to increase along with tumour progression (Figure 7D). Intriguingly, Kaplan–Meier curve analysis demonstrated that higher ALDH1A1 expression levels were correlated with reduced overall survival duration in LUSC patients (Figure 7E). These results suggest that ALDH1A1 predicts poor prognosis in LUSC patients.

## 3 DISCUSSION

Chronic chromium exposure induces human LUSC.^2^, ^3^ Due to its water‐soluble property, Cr(VI) is one of the most toxic heavy metal species.^4^ Current knowledge on the mechanism of Cr(VI)‐induced carcinogenesis involves DNA damage induced by excessive ROS produced by metabolic reduction of Cr(VI) to Cr(III), as well as by Cr(III)–DNA crosslinking.^27^ Here, we present an unappreciated mechanism underlying Cr(VI)‐transformed LUSC, in which Cr(VI) induces expression of ALDH1A1, a marker of CSCs/TICs, through KLF4 in Cr(VI)‐transformed epithelial cells (CrT). The Cr(VI)‐induced ALDH1A1 maintains self‐renewal of the CrT/TIC subpopulation and promotes expression and secretion of EGF from CrT/TICs to activate EGFR signalling of differentiated cancer cells, promoting LUSC tumourigenesis (Figure 7F). CSCs/TICs contribute to tumour initiation and recurrence. The finding that Cr(VI) exposure leads to the formation of an ALDH1A1‐positive TIC subpopulation elucidates another layer of Cr(VI)‐induced LUSC tumourigenesis in addition to DNA damage.

Gemcitabine is a nucleoside analogue with an anti‐LUSC activity that is used for first‐line LUSC treatment. However, chemotherapeutic resistance limits its clinical effectiveness.^28^ Targeting CSCs/TICs has been shown to benefit the tumour therapy.^29^ In particular, a subset of CSCs with specific metabolic signatures in pancreatic ductal adenocarcinoma (PDAC) displays strong gemcitabine resistance.^30^ These CSCs confer gemcitabine resistance to differentiated cancer cells through extracellular vesicles containing variant “resistance‐related cargo”.^31^ Targeting this subset of cancer cells has beneficial effects on the clinical therapy.^32^ Pancreatic CSCs have been reported to weaken the tumouricidal effect of gemcitabine treatment, which can be abolished by autophagy blockade.^33^ Moreover, inhibition of the CSC‐dependent glycosyltransferase ST6Gal‐I also impairs CSC activity, enhancing gemcitabine sensitivity.^34^ However, little is known about the roles of CSCs and related treatment strategies in LUSC. In the present study, we found that ALDH1A1 maintains the CSC phenotype in Cr(VI)‐induced LUSC and that targeted inhibition of ALDH1A1 by A37 increased the sensitivity of LUSC to gemcitabine treatment.

Collectively, our study suggests that a Cr(VI)‐induced CSC‐like subpopulation with high ALDH1A1 activity is the driving force for LUSC formation. Further, we found that the KLF4/ALDH1A1/EGF regulatory axis contributes to Cr(VI)‐induced carcinogenesis and promotes cancer cell differentiation. ALDH1A1 may have diagnostic value and serve as a novel therapeutic target.

## 4 MATERIALS AND METHODS

### 4.1 Materials

Antibodies against KLF4 (sc‐166238), DACH1 (sc‐398706), ABCB5 (sc‐517210), and SOX2 (sc‐365823) were purchased from Santa Cruz Biotechnology (CA, USA). Antibodies against ALDH1A1 (36671S), pan AKT (4685S), AKT pT308 (13038S), EGFR (4267S), EGFR pY1068 (3777S), Ki‐67 (9449S), RARα (62294), and H3K27ac (8173) were purchased from Cell Signaling Technology (MA, USA). Antibodies against CD133 (18470‐1‐AP) and CD44 (15675‐1‐AP) were purchased from Proteintech (Wuhan, China). Antibodies against GAPDH were purchased from Sigma–Aldrich (Shanghai, China). The antibody against ERK1/2 pT202/Y304 (AF1015) was purchased from Affinity Biosciences (Jiangsu, China). The antibody against MERTK (AF1015) was purchased from Thermo Fisher Scientific (MA, USA). *DACH1* siRNA (sc‐77089), *ABCB5* siRNA (sc‐89856), *MERTK* siRNA (sc‐37127), *KLF4* siRNA (sc‐35480), *SOX2* siRNA (sc‐38408), *EGF* siRNA (sc‐39416), and *ALDH1A1* siRNA (sc‐41442) were purchased from Santa Cruz Biotechnology (CA, USA). K_2_Cr_2_O_7_ (207802), 2‐hydroxyethyl agarose (A9045), NAC (A7250), DCFH‐DA (287810), A37 (531726000), Tyrphostin AG 1478 (T4182), RA (R2625), and 3dGRO Basal Medium (S3077A) were purchased from Sigma–Aldrich (MA, USA). Complete Protease Cocktail was purchased from Roche. Doxycycline was purchased from Selleckchem (MA, USA).

### 4.2 *In* *vivo* assay

BALB/c nude mice were purchased from qualified suppliers and fed under specific pathogen‐free conditions. To establish the subcutaneous tumourigenesis model, certain amounts of indicated cells mixed with Matrigel (Corning, NY, USA) were subcutaneously injected into the left flank. To establish the lung orthotopic tumourigenesis model, indicated cells mixed with Matrigel were injected into the left lung.

### 4.3 Immunohistochemical staining

Paraffin‐embedded tumour slides were immunostained with Ki‐67, CD44, CD133, ALDH1A1, and EGFR pY1068 antibodies. The staining outcome was quantitatively scored according to the staining intensity and the positive cells percentage.^35^

### 4.4 Cell culture and transfection

BEAS‐2B, HCC95, and H226 cells were obtained from American Type Culture Collection and cultured according to the protocol provided by ATCC. CrT cells were Cr(VI)‐induced malignant transformed cells; CrT/TICs and HCC95/TICs were selected and cultured with 3dGRO Basal Medium. Cells were transfected at 70%–80% confluence as previously described.^36^

### 4.5 Selection of tumour‐initiating cells

BEAS‐2B, CrT, and HCC95 cells were passaged in 3dGRO™ Spheroid Medium. Suspended cells were passaged once per week for several generations according to the standard protocol. Generally, after 10 passages, TICs were successfully selected and enriched as the tumoursphere, a cellular spheroid with low light transmittance.

### 4.6 ALDEFLUOR assay and flow cytometry

Cells were digested and suspended in ALDEFLUOR assay buffer and incubated following the standard instructions. Cells incubated with the ALDH inhibitor DEAB (15 μM) were used as a negative control to draw the gate for the experimental group. The relative intensity of the FITC from ALDH1A1‐positive cells was tested by flow cytometry. The top 10% and bottom 10% based on the ALDH enzymatic activity were sorted and designated as ALDH1A1^High^ and ALDH1A1^Low^, respectively.

### 4.7 ALDH activity measurement

The ALDH activity kit (#MAK082, Sigma–Aldrich, USA) was used to determine the ALDH activity. CrT/TICs were incubated with pre‐chilled ALDH assay buffer and incubated with the appropriate reaction buffer containing ALDH substrate mix and acetaldehyde. OD value (450 nm) was measured by microplate reader.

### 4.8 ChIP assay

The ChIP assay was performed using a ChIP assay kit (9005) from Cell Signaling Technology (Beverly, MA, USA). Briefly, 4 × 10^6^ cells were fixed and then lysed. Chromatin was digested into DNA fragments by micrococcal nuclease. Uncrosslinked DNA was purified using a DNA purification centrifuge column. Indicated antibodies were used to enrich DNA fragments. qRT‐PCR was performed using designed primers (Table S2).

### 4.9 Luciferase assays

JASPAR (https://jaspar.genereg.net/) was used to check the genomic regulatory elements related to the mentioned transcription factors. Furthermore, the potential *ALDH1A1* and *EGF* transcriptional regulation regions were cloned into the pGL3‐Basic vector. The luciferase activity was measured following the standard instructions.

### 4.10 Reactive oxygen species detection

Cells were detached with trypsin and collected after centrifugation. Then incubated with DCFH‐DA in dark and washed two times. DCF fluorescence was detected by flow cytometry (excitation wavelength: 488 nm; emission wavelength: 525 nm.)

### 4.11 Measurement of the NAD+/NADH ratio

After indicated treatments, ALDH1A1^High^ and ALDH1A1^Low^ CrT cells (1 × 10^6^ cells per sample) were harvested. Cells were lysed and centrifuged. The supernatant was determined the total NAD^+^/NADH levels (NAD_total_) first. To measure NADH levels, the supernatant was incubated at 60°C to thoroughly remove NAD^+^. Next, all samples were mixed and incubated with alcohol dehydrogenase. Then, the chromogenic solution was added. The OD values: 450 nm; the calculation formula: [NAD^+^]/[NADH] = ([NAD_total_] − [NADH])/[NADH].

### 4.12 PCR arrays

RT^2^ Profiler™ PCR Arrays (Qiagen) were used to analyse the difference between BEAS‐2B and CrT cells in terms of stem cell characteristics. Significantly differentially expressed genes are listed in Table S1. Multiplex PCR‐based preamplification of the pooled RNA samples of BEAS‐2B and CrT cells was performed using the RT^2^ Nano PreAMP cDNA Synthesis Kit (Qiagen).

### 4.13 Intracellular retinoic acid analysis

RA levels were detected using a human RA ELISA kit (CUSABIO, Wuhan, China). Cells were lysed on ice, the lysate was centrifuged, and the supernatant was collected. Samples or standards were added into 96‐well plates and mixed with TMB substrate. The OD value was detected at 450 nm. Finally, the RA concentration was calculated.

### 4.14 Statistical analysis

All data are shown as the mean ± standard deviation (SD) from triplicate replications. Two‐tailed unpaired Student's *t*‐test was performed for statistical analyses. *P* < .05 was considered statistically significant.

## AUTHORs’ CONTRIBUTIONS

Xu Qian, Bing‐Hua Jiang, Ling‐Zhi Liu, and Hua Shen conceived and designed the study and interpreted the results. Xin Ge, Mengdie Li, Zhixiang Zhang, Jianxing Yin, Zehe Ge, and Zhumei Shi performed most of the experiments. Hua Shen and Guo‐Xin Song collected and analysed clinical samples. Xu Qian wrote the manuscript with comments from all authors.

## CONFLICT OF INTERESTS

The authors declare that there is no conflict of interest that could be perceived as prejudicing the impartiality of the research reported.

## Supporting information

Supplementary Information

Supplementary Information

Supplementary Information

Supplementary Information
