# Supplementary figures and images for: Chromium (VI)‐induced ALDH1A1/EGF axis promotes lung cancer progression
Source: Clin Transl Med. 2022 Dec 11;12(12):e1136. doi: 10.1002/ctm2.1136 (PMC9742488; doi:10.1002/ctm2.1136)

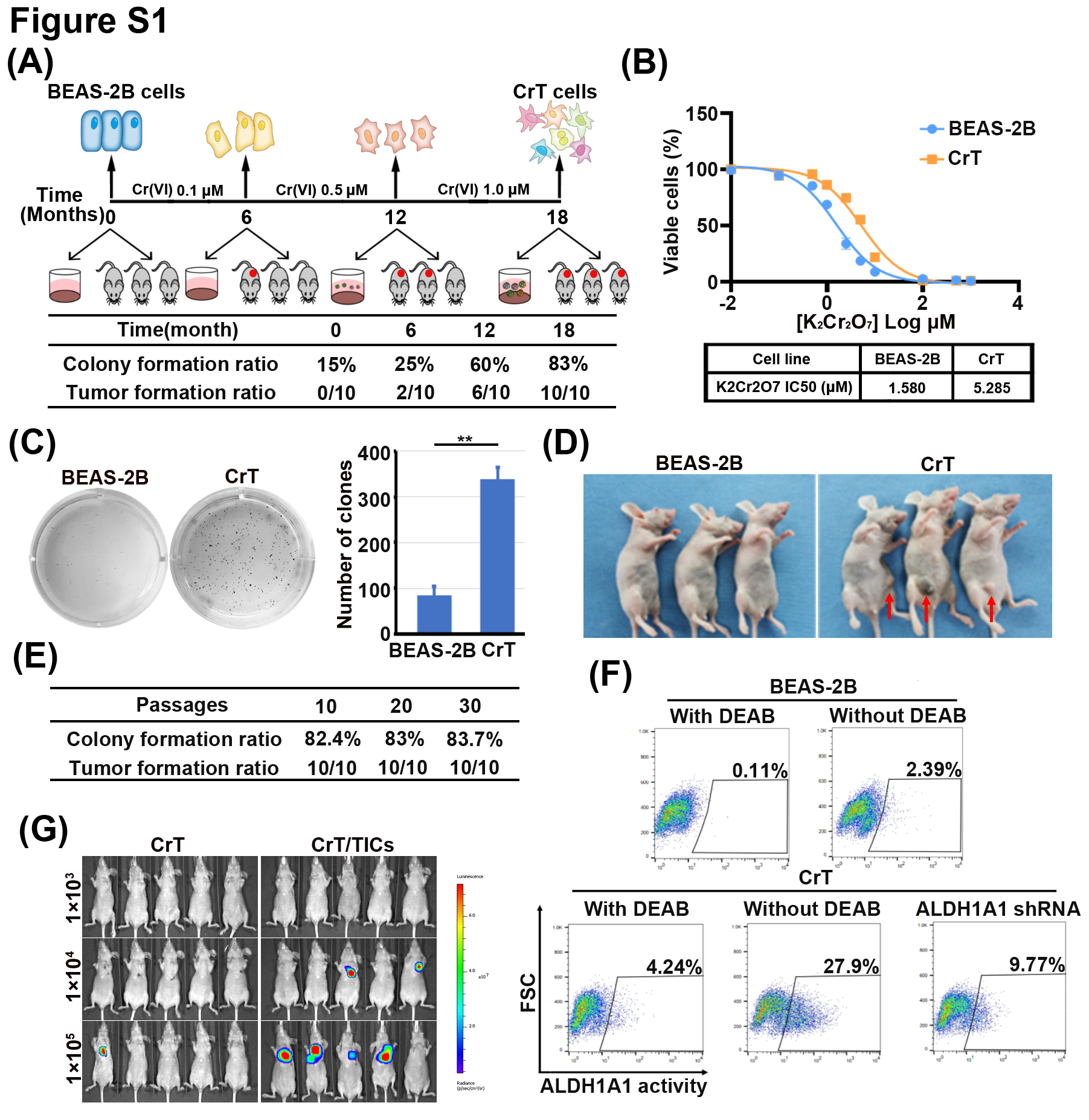

Supplement: Supplementary file 1 — Supplementary Information [file CTM2-12-e1136-s001.tif]
