# Supplementary material for: Chromium (VI)‐induced ALDH1A1/EGF axis promotes lung cancer progression
Source: Clin Transl Med. 2022 Dec 11;12(12):e1136. doi: 10.1002/ctm2.1136 (PMC9742488; doi:10.1002/ctm2.1136)
Supplement: Supplementary file 2 — Supplementary Information [file CTM2-12-e1136-s003.docx]

**SUPPLEMENTARY INFORMATION**

**Supplementary Figure 1 Establishment of Cr(VI)-transformed lung epithelial cells (CrT).**

1. Scheme of Cr(VI)-induced malignant transformation of lung epithelial cells. The transformed cells were termed CrT.
2. Dose-response curves for Cr(VI) in BEAS-2B and CrT cells.
3. Representative images of anchorage-independent colony formation derived from BEAS-2B and CrT cells (left). Data represent the mean ± SD of triplicate experiments (right). ***p* < 0.001
4. Images of mice bearing the subcutaneous tumors formed by BEAS-2B cells and CrT cells, respectively.
5. Colony formation ratio and tumor formation ratio by CrT cells of passage 10, 20, and 30.
6. ALDH1A1 activity was detected by flow cytometry in BEAS-2B or CrT cells with or without ADLH1A1 depletion.
7. Representative BLIs of orthotopic tumorigenesis generated by the indicated amount of CrT and CrT/TICs cells.
